# Antimicrobial resistance in coagulase-positive staphylococci isolated from companion animals in Australia: A one year study

**DOI:** 10.1371/journal.pone.0176379

**Published:** 2017-04-21

**Authors:** Sugiyono Saputra, David Jordan, Kate A. Worthing, Jacqueline M. Norris, Hui S. Wong, Rebecca Abraham, Darren J. Trott, Sam Abraham

**Affiliations:** 1 Australian Centre for Antimicrobial Resistance Ecology, School of Animal and Veterinary Sciences, The University of Adelaide, Roseworthy, SA, Australia; 2 Research Center for Biology, Indonesian Institute of Sciences, Cibinong, West Java, Indonesia; 3 New South Wales Department of Primary Industries, Wollongbar, NSW, Australia; 4 Faculty of Veterinary Science, The University of Sydney, Camperdown, NSW, Australia; 5 School of Veterinary and Life Sciences, Murdoch University, Perth, WA, Australia; Kent State University, UNITED STATES

## Abstract

Methicillin-resistant coagulase-positive staphylococci (CoPS) have become increasingly recognised as opportunistic pathogens that limit therapeutic options in companion animals. The frequency of methicillin resistance amongst clinical isolates on an Australia-wide level is unknown. This study determined antimicrobial susceptibility patterns for CoPS isolated from clinical infections in companion animals (dogs, cats and horses) as part of the first nation-wide survey on antimicrobial resistance in animal pathogens in Australia for a one-year period (January 2013 to January 2014). Clinical *Staphylococcus* spp. isolates (n = 888) obtained from 22 veterinary diagnostic laboratories were identified by MALDI-TOF mass spectrometry and subjected to antimicrobial susceptibility testing for 16 antimicrobials, representing 12 antimicrobial classes. Potential risk factors associated with methicillin resistance in *Staphylococcus pseudintermedius* isolates from dogs were analysed based on demographic factors and clinical history, including gender, age, previous antimicrobial treatment, chronic and/or recurrent diseases and site of infections. The most commonly identified CoPS were *S*. *pseudintermedius* (70.8%; dogs n = 616, cats n = 13) and *S*. *aureus* (13.2%, horses n = 53, dogs n = 47 and cats n = 17). Overall, the frequency of methicillin resistance among *S*. *pseudintermedius* (MRSP) and *S*. *aureus* (MRSA) was 11.8% and 12.8%, respectively. MRSP isolates were strongly associated with resistance to fluoroquinolones (OR 287; 95%CI 91.2–1144.8) and clindamycin (OR 105.2, 95%CI 48.5–231.9). MRSA isolates from dogs and cats were also more likely to be resistant to fluoroquinolones (OR 5.4, 95%CI 0.6–252.1), whereas MRSA from horses were more likely to be resistant to rifampicin. In multivariate analysis, MRSP-positive status was significantly associated with particular infection sites, including surgical (OR 8.8; 95%CI 3.74–20.7), and skin and soft tissue (OR 3.9; 95%CI 1.97–7.51). *S*. *pseudintermedius* isolated from dogs with surgical site infections were three times more likely to be methicillin-resistant if cases had received prior antimicrobial treatment. Whilst the survey results indicate the proportion of CoPS obtained from Australian companion animals that are methicillin-resistant is currently moderate, the identified risk factors suggest that it could rapidly increase without adequate biosecurity and infection control procedures in veterinary practice.

## Introduction

Coagulase-positive staphylococci (CoPS) cause a range of infections such us bacteraemia, urinary tract infections, pyoderma, abscess and wound infections in both humans and animals [1]. Infections are compounded by the emergence of methicillin-resistant strains that have acquired *mecA* or *mecC* imparting resistance to all the beta-lactams with the exception of a few anti-staphylococci cephalosporins [2]. The major methicillin-resistant CoPS that cause clinical infections are methicillin-resistant *Staphylococcus aureus* (MRSA) in both humans and animals [3], and methicillin-resistant *S*. *pseudintermedius* (MRSP) in dogs and cats [4]. MRSA and MRSP isolates are often resistant to multiple classes of critically important antimicrobials (CIAs) including fluoroquinolones and aminoglycosides, thereby limiting therapeutic options to treat these infections. In recent years, studies have demonstrated the emergence and clonal spread of MRSA in companion animals (defined here as dogs, cats and horses), and livestock, with potential for bi-directional transmission of these strains between animals and humans [5–7].

In companion animals, distinct MRSA clones appear to colonise specific animal host species. For example, healthcare associated MRSA clone ST22 (EMRSA-15) is most commonly isolated from dogs and cats while community associated MRSA CC 8 (ST8, ST612 and ST254) clones are host-adapted to horses [8]. A recent study by Harrison et al. has also demonstrated that globally disseminated MRSA ST22-IV strains can colonise and cause infection in humans, dogs, and cats without undergoing typical host adaption involving loss or acquisition of antimicrobial resistance and/or prophage genes [9]. These studies demonstrate the need for understanding the ecology and distribution of MRSA clones in companion animals.

Parallel to the emergence of MRSA in companion animals, MRSP has recently emerged in dogs and cats as a cause of skin and soft tissue, post-surgical site and urinary tract infections. Globally, the frequency of MRSP infections in dogs and cats has been increasing and MRSP is now considered to be one of the most important pathogens in small animal medicine [10]. This is attributed to the global spread of MRSP clones and the associated resistance to other CIAs such as fluoroquinolones and aminoglycosides. Unlike MRSA in companion animals, MRSP is not a major zoonotic pathogen and has limited public health impact [10]. However, due to the limited therapeutic options to treat MRSP infections they are now a major animal health issue and require careful monitoring and management [11].

Various studies have demonstrated carriage and zooanthroponotic transmission of MRSA and other multidrug-resistant staphylococci [12] between animals and humans. Consequently, many countries have established surveillance programs to monitor emerging antimicrobial resistance in animals, although companion animals are generally poorly represented in these activities. The frequency and antimicrobial resistance profile of clinical staphylococci in companion animals has been reported in Asia [13, 14], Africa [15], North America and Europe [16]. Sweden and Norway are among the few countries that monitor the occurrence of these resistant strains regularly [17, 18], enabling more accurate estimation of frequency, trends and antimicrobial resistance profiles to be compared on a yearly basis.

In Australia, several one-off studies have documented the recent emergence of MRSA and MRSP as causes of clinical infections as well as carriage by healthy companion animals [11, 19–21]. In addition, carriage of MRSA by Australian veterinarians involved in clinical practice has been well documented with the highest rates of carriage in equine veterinarians (21.4%), mixed-practice veterinarians (11.8%) and those who work exclusively with dogs and cats (4.9%) [7, 19, 22]. However, the frequency of methicillin resistance amongst isolates from infections in companion animals on an Australia-wide level is currently unknown. Therefore, in the present study, we undertook the first national survey of antimicrobial resistance in CoPS isolated from clinical infections in Australian companion animals. The aims were to define the distribution of CoPS species causing clinical infection in companion animals, the frequency of antimicrobial resistance (particularly methicillin resistance and multidrug resistance) and to examine potential risk factors that may contribute to the occurrence of methicillin-resistant strains amongst the most prevalent species.

## Materials and methods

### Isolate collection and identification

The CoPS isolates were collected during the first national survey of antimicrobial resistance in Australian animal pathogens, which took place over 12 months (January 2013 to January 2014) with the cooperation of all veterinary diagnostic laboratories in each Australian state and territory (n = 22) [23]. Submitting laboratories were instructed to forward coagulase-positive isolates that were considered to be clinically relevant to the presenting condition, as judged by the diagnostic microbiologist. The bacteria were isolated from swabs taken from site of infections or clinical specimens (e.g. urine, biopsies) collected by veterinarians and submitted to veterinary diagnostic laboratories for routine culture and susceptibility testing. All confidential information such as animal name, owner name, address and contract information was removed by the participating veterinary diagnostic laboratories before sending the isolates and clinical information to The University of Adelaide reference laboratory for this study. As a result this study did not require animal ethics approval, as per the Australian National Health and Medical Research Council, Animal Research Ethics code.

Prior to cryopreservation in 20% glycerol broth, isolates were confirmed for purity and haemolysis pattern on Columbia sheep blood agar (SBA; Thermo Fisher Scientific Australia), and identified to genus level using standard phenotypic tests including Gram-stain and the catalase test. A total of 888 isolates were collected in this study, originating from dogs (n = 743, 83.7%), cats, (n = 77, 8.7%) and horses (n = 68, 7.7%). To confirm the identity of staphylococci isolates to species level, all isolates were subjected to MALDI-TOF (Bruker) according to the manufacturer’s protocol for bacterial identification.

### Antimicrobial susceptibility testing and interpretation

Minimum inhibitory concentrations (MICs) were determined by the broth microdilution method of the Clinical Laboratory Standards Institute (CLSI) [24] (Table 1). A total of 16 antimicrobial agents from 12 antimicrobial classes were investigated including aminoglycosides (AMK); ansamycins (RIF); β-lactam/β-lactamase inhibitor combinations (AMC); β-lactams (OXA); fluoroquinolones (CIP, ENR, MRB and PRA); folate-pathway inhibitors (SXT); 1^st^ generation cephalosporins (CEF); 2^nd^ generation cephamycin (FOX); 3^rd^ generation cephalosporins (CVN and CRE); lincosamides (CLI); phenicols (CHL); and tetracyclines (TET). Antimicrobials were obtained from Sigma Aldrich (Australia) and Zoetis (Australia). *Staphylococcus aureus* ATCC 25923 and ATCC 29213 were used as control strains. MIC results were interpreted as resistant (R), susceptible (S) and intermediate (I, if available), according to veterinary specific and human approved interpretative criteria per Clinical and Laboratory Standards Institute (CLSI) VET01S guidelines [25]. When clinical breakpoints were not available in CLSI, MICs were interpreted based on epidemiological cut-off values (ECOFFs) as non-wild type (non-WT) organisms derived from assessment of the MIC distribution using ECOFFinder [26, 27] and/or as published by European Committee on Antimicrobial Susceptibility Testing (EUCAST) [28] as presented in Table 1.

**Table 1:** Antimicrobial agents and MIC breakpoints (μg/mL) used in this study based on CLSI VET01S and ECOFFs criteria.

Antimicrobial agent	Code	*S*. *pseudinternedius*	*S*. *aureus*
Amikacin	AMK	≥16	≥16
Amoxicillin-clavulanate	AMC	≥1/0.5; ≥16/8	≥1/0.5; ≥16/8
Cefovecin	CVN	≥1	≥4
Cefoxitin	FOX	≥1	≥8
Ceftriaxone	CRE	≥4	≥16
Cephalothin	CEF	≥0.5	≥8
Chloramphenicol	CHL	≥32	≥32
Ciprofloxacin	CIP	≥4	≥4
Clindamycin	CLI	≥4	≥4
Enrofloxacin	ENR	≥4	≥4
Marbofloxacin	MRB	≥4	≥4
Oxacillin	OXA	≥0.5	≥4
Pradofloxacin	PRA	≥2	≥2
Rifampicin	RIF	≥4	≥4
Tetracycline	TET	≥1	≥1
Trimethoprim-sulfamethoxazole	SXT	≥4/76	≥4/76

For *S*. *pseudintermedius*, veterinary specific breakpoints were used for AMK, AMC, CLI, ENR, MRB, PRA and TET; human interpretative criteria were used for CHL, CIP, OXA, RIF and SXT and ECOFF criteria as defined by ECOFFinder were used for CVN, FOX, CRE and CEF. For *S*. *aureus*, veterinary specific breakpoints were used for AMK, AMC, CEF, CLI, ENR, MRB, PRA and TET; human interpretative criteria were used for FOX, CHL, CIP, OXA, RIF and SXT; ECOFF criteria were used for CVN (defined by ECOFFinder) and CRE (defined by EUCAST). In this study, we used breakpoints for CEF of ≥0.5 μg/mL instead of ≥8 μg/mL for *S*. *pseudintermedius* as stated in CLSI VET01S in order to correspond with ECOFF criteria and presence of *mecA* genes in the isolates. Also, for dog and horse isolates, the veterinary specific breakpoint for AMC of ≥1/0.5 μg/mL was used for isolates from skin and soft tissue infections (SSTIs) and the breakpoint ≥16/8 μg/mL (non-susceptible) was used for isolates from urinary tract infections (UTIs). For cat isolates, a breakpoint for AMC of ≥1/0.5 μg/mL was used for both SSTIs and UTIs.

Isolates showing resistance to three or more antimicrobial classes interpreted by clinical breakpoints were classified as multidrug-resistant (MDR) [29]. The frequency of antimicrobial resistance according to established breakpoints were described as rare: <0.1%; very low: 0.1% to 1.0%; low: >1% to 10.0%; moderate: >10.0% to 20.0%; high: >20.0% to 50.0%; very high: >50.0% to 70.0%; and extremely high: >70.0%; according to the European Food Safety Authority (EFSA) and the European Centre for Disease Prevention and Control (ECDC) [30].

### Confirmation of methicillin resistance status

Phenotypic confirmation of methicillin resistance status for putative MRSA and MRSP strains was assessed using resistance to cefoxitin and/or oxacillin, as well as colony appearance on Brilliance™ Agar MRSA2 (Thermo Fisher Scientific, Australia). Additionally, *mecA* PCR [31] was undertaken on all *S*. *aureus* isolates with cefoxitin MICs ≥8 μg/mL and all *S*. *pseudintermedius* isolates with oxacillin MICs ≥0.5 μg/mL as recommended by CLSI VET01S [25]. Methicillin-resistant staphylococci were reported as resistant to all penicillins, cephems and β-lactams/β-lactamase inhibitor combinations regardless of *in vitro* test results with those agents [24].

### Risk factor analysis

Of the 794 CoPS isolates analysed in this study, a total of 661 (dogs n = 597, 90.3%; cats n = 16, 2.4%; horses n = 35, 5.3%) were accompanied by a detailed clinical history. However, due to low sample size, only *S*. *pseudintermedius* isolates from dogs (n = 555) were further interrogated in the risk factors study. *S*. *pseudintermedius* that were methicillin-resistant (n = 68) were used as the outcome in this analysis. The variables for potential risk factors were gender (male or female), age group (<2 years, 2–10 years or >10 years), previous antimicrobial treatment (yes/ no), chronic and/or recurrent diseases (yes/ no), and site of infection (ear, urinary tract, skin and soft tissue, surgical site or respiratory tract). Initially, univariate analyses were used to assess the effect of various factors on the frequency of methicillin resistance in *S*. *pseudintermedius* isolates from dogs. This was followed by construction of a multivariate logistic regression model to account for the possible effects of confounding and interaction. Age of animal was forced into the multivariate model as a probable confounder and then each explanatory variable was assessed for its significance on the outcome. The most significant explanatory variables were then added to the model and the process repeated (by adding only significant variables) to obtain a main effects model. Two-way interactions between the main effects variables were then explored and retained when significant at P<0.05. Statistical analyses were performed using Stata/MP 14.0 (Stata Corp., College Station, TX, USA).

## Results

### Distribution of staphylococci species

Of the 888 isolates from companion animals submitted by Australian veterinary diagnostic laboratories for this study, a total of 877 isolates (98.8%) were confirmed to belong to the *Staphylococcus* genus. The most commonly identified CoPS were *S*. *pseudintermedius* (n = 629) and *S*. *aureus* (n = 117). Other CoPS identified included *S*. *schleiferi* (n = 44), *S*. *intermedius* (n = 2) and *S*. *delphini* (n = 2). Of the 629 *S*. *pseudintermedius* isolates, 97.9% were obtained from dogs and 2.1% from cats. Of the 117 *S*. *aureus* isolates, 45.3% were recovered from horses, 40.1% from dogs, and 14.5% from cats. All *S*. *schleiferi* isolates originated from dogs while one *S*. *intermediu*s was isolated from a dog and a cat, respectively. Both *S*. *delphini* isolates came from horses. A small number of coagulase-negative staphylococci were also identified, as either *Staphylococcus felis* (n = 34) isolated from cats, *Staphylococcus epidermidis* (n = 10) from dogs and cats and *Staphylococcus sciuri* (n = 10) from horses and dogs. Coagulase-negative isolates were excluded from further analyses.

### Phenotypic antimicrobial resistance among *S*. *pseudintermedius*

The MIC distribution and frequency of antimicrobial resistance among *S*. *pseudintermedius* is shown in Table 2. Among 81 isolates with oxacillin MICs ≥0.5 μg/mL, a total of 74 isolates (11.8%, dog n = 72, cat n = 2) were classified as methicillin-resistant *S*. *pseudintermedius*. The remaining isolates (n = 7) were classified as methicillin-susceptible because of negative results either on the basis of *mecA* PCR and colony appearance on Brilliance™ MRSA 2 Agar (S1 Table).

**Table 2:** MIC distribution and frequency of resistance (%R) among clinical *Staphylococcus pseudintermedius* isolated from dogs (n = 616) and cats (n = 13) in Australia^a^.

Antimicrobials	Animals	% R	95% CI	Percentage of isolates with indicated MIC^b^														
				≤0.004	0.008	0.016	0.03	0.06	0.13	0.25	0.5	1	2	4	8	16	32	≥64
Amikacin	Dog	1.1	0.5–2.3								1.3	10.7	59.1	22.6	5	0.8		0.3
	Cat	0	0.0–24.7									23.1	53.8	15.4	7.7			
Amoxicillin-clavulanate	Dog (SSTI)	45.1	40.7–49.6					3.4	15.3	12.1	24.1	18.7	14.1	1	1.8	1.6	8	
	Dog (UTI)	3.6	1.1–9.4					2.7	8.9	14.2	26.6	30.1	13.3		0.9	0.9	2.7	
	Cat	53.8	26.7–80.9					7.7	23.1	7.7	7.7	23.1	7.7			7.7	15.4	
Cefovecin	Dog	13.1	10.6–16.1					2.8	71.8	11.5	0.6	3.1	0.6	0.3		0.2	0.2	8.8
	Cat	15.4	1.9–45.4						61.5	15.4	7.7							15.4
Cefoxitin	Dog	11.5	9.1–14.3					1.3	36.2	40.1	10.7	3.7	3.6	1.9	1.5	0.2	0.5	0.2
	Cat	23.1	5.0–53.8						46.2	23.1	7.7	7.7	7.7				7.7	
Ceftriaxone	Dog	12.8	10.3–15.7				0.2	0.2	0.5	1	37.2	46.9	1.1	2.1	1.1	0.8	8.8	
	Cat	23.1	5.0–53.8								23.1	53.8		7.7			15.4	
Cephalothin	Dog	13.5	10.9–16.4					28.4	50.5	7.5	2.9	1	0.5	0.6	1.5	0.8	1.9	4.2
	Cat	23.1	5.0–53.8					15.4	61.5			7.7			7.7	7.7		
Chloramphenicol	Dog	5.7	4.0–7.8						0.2				0.5	54.1	39	0.6	0.2	5.5
	Cat	7.7	0.2–36.0											38.5	53.8			7.7
Ciprofloxacin	Dog	8.1	6.1–10.6			0.2	0.5	12	61.5	12.7	2.9	1.3	0.6	1	7.1			
	Cat	0.0	0.2–36.0					23.1	53.8	15.4		7.7						
Clindamycin	Dog	12.7	10.1–15.5				0.2	10.7	69	6.3	0.5	0.3	0.2	0.5	0.6		11.5	
	Cat	7.7	0.2–36.0					7.7	84.6								7.7	
Enrofloxacin	Dog	8.1	6.1–10.6			0.2	0.6	15.9	56.3	12.3	3.6	2.1	0.6	0.8	7.3			
	Cat	0	0.0–24.7					15.4	69.2	15.4								
Marbofloxacin	Dog	8.8	6.7–11.3				0.5	0.2	5.4	61	19.2	4.5	0.3	1.3	7.5			
	Cat	0	0.0–24.7							84.6	15.4							
Oxacillin	Dog	12.7	10.1–15.5				0.2	2.1	61.2	23.7	1.5	0.8	0.8	0.8	0.3	0.3	1.3	6.8
	Cat	23.1	5.0–53.8						76.9		7.7							15.4
Pradofloxacin	Dog	6.5	4.7–8.7			5.7	38.8	42	2.9	1.5	1.3	1.1	5.7	0.8	0.2			
	Cat	0	0.0–24.7				46.2	38.5	7.7	7.7								
Rifampicin	Dog	1	0.1–2.1	37	58.9	2.9			0.2					1				
	Cat	0	0.0–24.7	38.5	61.5													
Tetracycline	Dog	22.7	19.5–26.2					18.7	52.1	6.2	0.3	0.2		0.2			11	11
	Cat	15.4	1.9–45.4					38.5	30.8	15.4								15.4
Trimethoprim-sulfamethoxazole	Dog	37.3	33.5–41.3					0.3	0.6	4.7	33	8.1	15.7	26.9	1.1	9.3		
	Cat	30.8	9.1–61.4							30.8	38.5			23.1		7.7		

^a^ Among dog isolates, SSTI n = 503, UTI n = 113, cat isolates SSTI n = 10, UTI n = 3.

^b^ Unshaded areas show the dilution range for each drug. Vertical solid lines indicate veterinary specific breakpoints.

Double vertical solid lines indicate human interpretative criteria. ECOFFs are indicated as vertical dotted lines. Resistance to CVN, FOX, CRE, CEF and OXA after confirmation of methicillin resistance status is presented in S2 Table.

Among dog isolates (n = 616), resistance to AMC (37.5% of isolates; 45.1% for SSTI, 3.5% for UTI) was most common followed by SXT (37.3%) and TET (22.7%). CLI resistance was observed at a moderate level (12.7%). Similarly, a moderate level of resistance was observed to fluoroquinolones, ranging from 6.5%-8.8% for the four compounds tested in this study (CIP, ENR, MRB, PRA). Resistance to CHL was observed in 5.7% of isolates. A very low number of isolates were resistant to AMK (1.1%; n = 7) and RIF (1%, n = 6).

Among cat isolates (n = 13), the most common resistance found was to AMC (53.8% of isolates) and SXT (30.8%). CHL and CLI resistance was detected in 7.7% isolates. Resistance to AMK, RIF and fluoroquinolones was not detected.

### Phenotypic antimicrobial resistance among S. *aureus*

The MIC distribution and frequency of antimicrobial resistance among *S*. *aureus* is shown in Table 3. Overall, 12.8% of the *S*. *aureus* isolates were methicillin-resistant, including six isolates from horses (11.3%), six isolates from dogs (12.8%), and three isolates from cats (17.6%). Among methicillin-susceptible *S*. *aureus*, resistance to at least one or more β-lactam antimicrobials was observed in three isolates (2.6%).

**Table 3:** MIC distribution and frequency of resistance (%R) among clinical *Staphylococcus aureus* isolated from horses (n = 53), dogs (n = 47), and cats (n = 17) in Australia^b^.

Antimicrobials	Animals	% R	95% CI	Percentage of isolates with indicated MIC^a^														
				≤0.004	0.008	0.016	0.03	0.06	0.13	0.25	0.5	1	2	4	8	16	32	≥64	
Amikacin	Horse	9.4	3.1–20.7							1.9	1.9	3.8	43.4	24.5	15.1	7.5	1.9		
	Dog	2.1	0.1–11.3							2.1	2.1	6.4	36.2	46.8	4.3	2.1			
	Cat	0	0.0–19.5										58.8	41.2					
Amoxicillin-clavulanate	Horse (SSTI)	47.2	33.7–60.5						9.4	22.6	20.8	17	3.8	9.4	3.8	1.9	11.3		
	Dog (SSTI)	59.5	43.3–74					7.1		14.3	19.1	9.5	14.3	19.1	2.4		14.3		
	Dog (UTI)	0	0–0.5							60		40							
	Cat	58.8	35.4–82.2					11.8		5.9	23.5	29.4	17.6				11.8		
Cefovecin	Horse	13.2	5.5–25.3						3.8	3.8	7.5	66	5.7	1.9				11.3	
	Dog	14.9	6.2–28.3					2.1	12.8	6.4	12.8	51.1			2.1			12.8	
	Cat	17.6	3.8–43.4							5.9	35.3	41.2						17.6	
Cefoxitin	Horse	11.3	4.3–23.0						3.8	3.8	1.9	3.8	43.4	32.1			5.7	5.7	
	Dog	12.8	4.8–25.7						10.6	10.6	2.1	4.3	38.3	21.3		2.1	2.1	8.5	
	Cat	17.6	3.8–43.4										29.4	52.9			5.9	11.8	
Ceftriaxone	Horse	13.2	5.5–25.3							1.9	1.9	9.4	15.1	58.5		1.9	11.3		
	Dog	12.8	4.8–25.7							2.1	8.5	12.8	17	46.8			12.8		
	Cat	17.6	3.8–43.4									5.9	52.9	23.5			17.6		
Cephalothin	Horse	11.3	4.3–23.0					5.7	3.8	35.8	32.1	9.4		1.9			5.7	5.7	
	Dog	12.8	4.8–25.7					12.8	12.8	8.5	38.3	14.9			4.3			8.5	
	Cat	11.8	1.5–36.4						5.9	17.6	41.2	17.6	5.9					11.8	
Chloramphenicol	Horse	1.9	0.0–10.1											15.1	83			1.9	
	Dog	2.1	0.1–11.3									2.1		25.5	70.2			2.1	
	Cat	0	0.0–19.5											5.9	94.1				
Ciprofloxacin	Horse	1.9	0.0–10.1					15.1	37.7	39.6	5.7				1.9				
	Dog	8.5	2.4–20.4					4.3	27.7	29.8	27.7	2.1			8.5				
	Cat	11.8	1.5–36.4							64.7	23.5				11.8				
Clindamycin	Horse	0	0.0–10.1					20.8	77.4		1.9								
	Dog	2.1	0.5–14.5				2.1	34	59.6		2.1						2.1		
	Cat	0	0.0–19.5					47.1	52.9										
Enrofloxacin	Horse	1.9	0.0–10.1				1.9	32.1	54.7	9.4					1.9				
	Dog	8.5	2.4–20.4				2.1	8.5	53.2	23.4	2.1	2.1			8.5				
	Cat	11.8	1.5–36.4					11.8	52.9	23.5					11.8				
Marbofloxacin	Horse	1.9	0.0–10.1					1.9	1.9	75.5	18.9				1.9				
	Dog	8.5	2.4–20.4					2.1	4.3	40.4	40.4	4.3			8.5				
	Cat	11.8	1.5–36.4							29.4	58.8				11.8				
Oxacillin	Horse	11.3	4.3–23.0						17	37.7	30.2	3.8					1.9	9.4	
	Dog	12.8	4.8–25.7					2.1	21.3	14.9	44.7	2.1	2.1				4.3	8.5	
	Cat	17.6	3.8–43.4						11.8	23.5	47.1			5.9				11.8	
Pradofloxacin	Horse	1.9	2.4–20.4			13.2	52.8	30.2			1.9		1.9						
	Dog	8.5	2.4–20.4			4.3	17	46.8	23.4					6.4	2.1				
	Cat	11.8	1.5–36.4			5.9	17.6	64.7					5.9	5.9					
Rifampicin	Horse	9.4	3.1–20.7	45.3	43.4	1.9								9.4					
	Dog	0	0.0–7.5	53.2	44.7			2.1											
	Cat	0	0.0–19.5	35.3	64.7														
Tetracycline	Horse	32.1	19.9–46.3						30.2	28.3	9.4		1.9		1.9		5.7	22.6	
	Dog	10.6	3.5–23.1					6.4	38.3	34	10.6		2.1				4.3	4.3	
	Cat	5.9	0.1–28.7						47.1	47.1								5.9	
Trimethoprim-sulfamethoxazole	Horse	13.2	5.5–25.3							50.9	30.2	3.8	1.9	1.9	3.8	7.5			
	Dog	6.4	1.3–17.5						2.1	44.7	38.3	4.3	4.3	4.3	2.1				
	Cat	0	0.0–19.5					5.9	5.9	52.9	35.3								

^a^ Among horse isolates, SSTI n = 53; dog isolates SSTI n = 42, UTI = 5; cat isolates SSTI n = 15, UTI n = 2.

^b^ Unshaded areas show the dilution range for each drug.

Vertical solid lines indicate veterinary specific breakpoints.

Double vertical solid lines indicate human interpretative criteria.

ECOFFs are indicated as vertical dotted lines.

Resistance to CVN, FOX, CRE, CEF and OXA after confirmation of methicillin resistance status is presented in S2 Table.

Among isolates from horses (n = 53), resistance to AMC and TET was high (47.1% and 32.1%, respectively). Resistance to SXT was observed in 13.2% of isolates and resistance to AMK and RIF in 9.4% of isolates. Resistance to CHL and fluoroquinolones was observed at a low level (1.9%). Resistance to CLI was not observed.

Among dog isolates (n = 47), AMC (57.4% of isolates; 59.5% for SSTI, 0% for UTI) and CVN (14.9%) had the highest rates of resistance. Resistance to fluoroquinolones was observed in 8.5% of isolates. A low frequency of resistance (2.1%) was observed for AMK and CHL.

Among cat isolates (n = 17), resistance to AMC was the most common (58.8%), followed by resistance to fluoroquinolones (11.8%). Resistance to five antimicrobials (AMK, CHL, CLI, RIF and SXT) was not detected.

### Resistance profiles of *S*. *pseudintermedius* isolates

The resistance profiles of the *S*. *pseudintermedius* isolates are presented in Table 4. In total, 51.2% of *S*. *pseudintermedius* isolates were fully susceptible to eight antimicrobial classes. The proportion of single drug resistance in *S*. *pseudintermedius* was 38.1%, with single SXT resistance the most common pattern (18.8%). MDR was observed in 83 isolates (13.2%) including 74 isolates that were regarded as MRSP based on phenotypic characteristics and *mecA* PCR and nine *S*. *pseudintermedius* isolates that were methicillin-susceptible. The most common MDR pattern was resistance to phenicols, lincosamides, fluoroquinolones (FQN), β-lactams, tetracycline and folate-pathway inhibitors (CHL-CLI-FQN-OXA-TET-SXT) in 23 canine MRSP isolates. Methicillin resistance was significantly associated with resistance to CLI (OR 105.2, 95%CI 48.5–231.9), FQN (OR 287; 95%CI 91.2–1144.8), TET (OR 7.5, 95%CI 4.4–13.1) and SXT (OR 8.5, 95%CI 4.6–16.6).

**Table 4:** Resistance profile per antimicrobial class found in clinical *Staphylococcus pseudintermedius* isolates in Australia (2013–2014)

Resistance profile^a^	No. (%) of isolates	
	Dog (n = 616)	Cat (n = 13)
0: NIL	316 (51.3)	6 (46.2)
1: CLI	6 (1)	-
1: FQN	1 (0.2)	-
1: OXA	7 (1.1)	2 (15.4)
1: TET	45 (7.3)	-
1: SXT	113 (18.8)	3 (23.1)
2: CHL-CLI	1 (0.2)	-
2: CLI-SXT	4 (6.5)	-
2: OXA-CLI	3 (0.5)	-
2: OXA-TET	1 (0.2)	-
2: OXA-SXT	7 (1.1)	-
2: FQN-OXA	1 (0.2)	
2: TET-SXT	46 (7.5)	1 (7.7)
3: CHL-TET-SXT	1 (0.2)	-
3: CLI-TET-SXT	3 (0.5)	-
3: OXA-CLI-SXT	3 (0.5)	-
3: OXA-TET-SXT	2 (0.3)	-
4: AMK-CLI-OXA-RIF	1 (0.2)	-
4: CLI-CHL-TET-SXT	1 (0.2)	-
4: CLI-OXA-RIF-TET	1 (0.2)	-
4: OXA-FQN-CLI-SXT	9 (1.5)	-
4: OXA-CLI-CHL-TET	4 (0.6)	-
5: AMK-CLI-FQN-OXA-SXT	5 (0.8)	-
5: CHL-CLI-FQN-OXA-TET	2 (0.3)	1 (7.7)
5: CHL-CLI-OXA-FQN-SXT	1 (0.2)	-
5: CHL-CLI-FQN-TET-SXT	1 (0.2)	-
5: CLI-FQN-OXA-TET-SXT	7 (1.1)	-
6: CHL-CLI-FQN-OXA-TET-SXT	23 (3.7)	-
6: OXA-FQN-CLI-RIF-TET-SXT	1 (0.2)	-
7: AMK-CLI-OXA-OXA-RIF-TET-SXT	1 (0.2)	-
7: CHL-CLI-OXA-FQN-RIF-TET-SXT	2 (0.3)	-
Total MRSP	72 (11.7)	2 (15.4)
Total MDR but not MRSP	9 (1.5)	-
Total MDR	81 (13.1)	2 (15.4)

^a^ Antimicrobial classes included: aminoglycosides (AMK); lincosamides (CLI), phenicols (CHL), fluoroquinolones (FQN, including CIP, ENR, MRB and PRA); β-lactams (OXA, representing methicillin resistance); ansamycin (RIF); tetracyclines (TET); and folate-pathway inhibitors (SXT). NIL, none.

### Resistance profiles of *S*. *aureus* isolates

The resistance profiles of *S*. *aureus* isolates are shown in Table 5. In total, 68.4% of *S*. *aureus* isolates were fully susceptible to eight antimicrobial classes. MDR was detected with a frequency of 12.8%, including six MRSA isolates from horses, six MRSA isolates from dogs and three MRSA isolates from cats. Resistance to TET was the most common pattern observed in horse isolates (17%) and dog isolates (6.4%). Dog and cat isolates were more likely to be resistant to fluoroquinolones (OR 5.4, 95%CI 0.6–252.1), which was also always associated with methicillin resistance, compared to horse isolates. Horse isolates were more likely to be amikacin-resistant (OR 6.5, 95%CI 0.7–315.2) compared to dog and cat isolates. All rifampicin-resistant *S*. *aureus* isolates from horses (n = 5) were methicillin-resistant.

**Table 5:** Resistance profile per antimicrobial class found in clinical *Staphylococcus aureus* isolates from horses, dogs and cats in Australia (2013–2014)

Resistance profile^a^	No. of isolates (%)		
	Horse (n = 53)	Dog (n = 47)	Cat (n = 17)
0: NIL	33 (62.3)	33 (70.2)	14 (82.3)
1: FOX	-	2 (4.2)	-
1: CHL	-	1 (2.1)	-
1: TET	9 (17)	3 (6.4)	-
1: SXT	2 (3.8)	2 (4.2)	-
2: AMK-TET	3 (5.7)	1 (2.1)	-
2: FOX-FQN	1 (1.9)	3 (6.4)	2 (11.8)
2: FOX-TET	-		1 (5.9)
2: TET-SXT	-	1 (2.1)	-
3: FOX-FQN-LNC	-	1 (2.1)	-
4: FOX-RIF-TET-SXT	2 (3.8)	-	-
5: AMK-FOX-RIF-TET-SXT	2 (3.8)	-	-
5: FOX-CHL-RIF-TET-SXT	1 (1.9)	-	-
Total MRSA	6 (11.3)	6 (12.8)	3 (17.6)
Total MDR	6 (11.3)	6 (12.8)	3 (17.6)

^a^ Antimicrobial classes included: aminoglycosides (AMK); 2^nd^ cephemycins (FOX, representing methicillin resistance); lincosamides (CLI), phenicols (CHL), fluoroquinolones (FQN, including CIP, ENR, MRB, PRA); ansamycin (RIF); tetracyclines (TET); and folate-pathway inhibitors (SXT). NIL, none.

### Risk factors for MRSP in dogs

In univariate analysis, there was no significant difference in the proportion of MRSP isolates between female versus male dogs; chronic versus non-chronically diseased dogs; or the various age groups (Table 6). Site of infection and prior antimicrobial treatment were significantly associated with MRSP isolation and were retained in the multivariate model. In multivariate analysis, after controlling for the confounding effect of age, isolates from particular infection sites, including surgical sites (OR 8.8; 95%CI 3.74–20.7), and skin and soft tissue (OR 3.9; 95%CI 1.97–7.51) continued to have a strong association with MRSP isolation. In the main effects model, prior antimicrobial treatment was not a significant factor contributing to the isolation of methicillin-resistant strains (OR 1.63; 95%CI 0.86–2.8). However, after inclusion of interaction terms, surgical site infections (OR 15.7; 95%CI 5.37–46.19) and skin and soft tissue infections (OR 6.1, 95%CI 2.52–14.84) were significantly more likely to be methicillin-resistant in dogs who had received prior antimicrobial treatment compared to dogs who had not received prior antimicrobial treatment (Table 7).

**Table 6:** Univariate analysis of risk-factor variables from *Staphylococcus pseudintermedius* isolates from dogs in Australia (n = 555). Odds ratios define the risk of isolates being classified as methicillin-resistant strains.

Risk factor	n	%MRSP	OR	P value	95% CI
Age in years					
<2	51	5.9	Ref		
2–10	391	13.3	2.45	0.143	0.74–8.17
<10	113	8.8	1.56	0.518	0.41–5.9
Chronic and recurrent disease					
No	492	12	Ref		
Yes	63	9.5	0.77	0.567	0.32–1.87
Prior antimicrobial treatment					
No	419	9.3	Ref		
Yes	136	19.1	2.3	0.002	1.34–3.95
Sex					
Male	247	11.3	Ref		
Female	308	12	1.07	0.805	0.563–1.8
Site of infection					
Ear	255	6.3	Ref		
Skin and soft tissue	138	19.6	3.63	<0.000	1.88–7.01
Urinary tract	104	5.7	0.91	0.865	0.34–2.4
Surgical site	42	35.7	8.3	<0.000	3.7–18.63
Respiratory tract	16	6.2	1	0.997	0.12–8.02

**Table 7:** Odds ratios showing the likelihood of isolates being methicillin-resistant in *Staphylococcus pseudintermedius* isolates from dogs in Australia for different combinations of site of infection in the host and exposure of the host to prior antimicrobial treatment.

Prior antimicrobial treatment^a^	Surgical site		Skin and soft tissue	
	n	OR; 95%CI	n	OR; 95%CI
No	19	5.4; 1.65–17.39	96	2.9; 1.32–6.45
Yes	23	15.7; 5.37-46-19	42	6.1; 2.51–14.84

^a^ Reference value isolates obtained from dogs with ear infections that did not receive prior antimicrobial treatment.

## Discussion

This is the first comprehensive study describing the distribution of antimicrobial susceptibility profiles in CoPS isolated from clinical infections in companion animals in Australia. This study generated three major findings: 1) The frequency of MRSP and MRSA isolation from clinical infections in companion animals in Australia was estimated as moderate (11.8% and 12.8% of total isolates for each species, respectively); 2) Resistance to critically important antimicrobials used in human medicine (fluoroquinolones, amikacin) remains very low to low among Australian companion animal CoPS; and 3) Prior antimicrobial treatment was identified as a significant risk factor for isolation of MRSP from dogs with surgical site, skin and soft tissue infections.

MRSP infections are increasingly reported in veterinary practice, spreading among companion animals and to a lesser extent among veterinarians [10]. The increased frequency of MRSP that are MDR poses a serious concern for biosecurity and infection control in veterinary practices, due to limited therapeutic options and the ease of transmission between animals. In parallel to other noteworthy studies from Australia [11], MRSP isolates were resistant to more antimicrobial classes than MRSA isolates, exemplified by the high proportion of MRSP isolates showing resistance to more than six antimicrobial classes (n = 29, 4.8%), while this level of multidrug resistance was not identified in any *S*. *aureus* isolates. Compared to similar surveys in other countries conducted over the same time period, the frequency of methicillin-resistant strains among canine *S*. *pseudintermedius* in Australia (11.8%) was significantly higher (P<0.0001) than that reported in Sweden (0.4%) [17] and Norway (0.5%) [18]. However, resistance to clindamycin in *S*. *pseudintermedius* in Australia (12.7%) was significantly lower (P = 0.0001) than in Sweden (21.6%). While a high level of amikacin resistance in *S*. *pseudintermedius* isolates has been demonstrated in some studies [32], we found that only a very low proportion of companion animal *S*. *pseudintermedius* isolates from Australia were resistant to this critically important human drug (n = 7; 1.1%). It is therefore recommended that use of amikacin in veterinary medicine continues to be reserved for MDR infections identified on the basis of culture and susceptibility testing when no other drug class is available [33].

In the only other comparable study conducted in 2006 in two regions of Australia, involving both clinical and non-clinical (i.e. carriage) of S*taphylococcus* spp. isolates from dogs and cats (n = 331), the frequency of methicillin-resistant (*mecA)* and β-lactam-resistant (*blaZ)* strains was only 3% and 6.9%, respectively [20, 34]. Although methodologies for sampling, testing and data interpretation were somewhat different to this study, it might indicate that methicillin resistance amongst Australian companion animal staphylococci has substantially increased in less than a decade.

In a recent Australian study, colonisation of veterinarians by MRSA was dominated by strains belonging to CC8 MRSA (ST8-IV [2B], *spa* t064; and ST612-IV [2B], *spa* variable). These were strongly associated with equine practice veterinarians and were often resistant to rifampicin and gentamicin [19]. MRSA CC8 (ST8 and ST612) is the most commonly identified clone among both Australian veterinarians and clinical equine samples [35]. Similarly, in the present study, a high proportion of MRSA isolates from cases of infection in Australian horses were also resistant to rifampicin (9.4%) but rarely resistant to fluoroquinolones. Rifampicin is almost exclusively used in equine practice, where it is combined with a macrolide for the oral treatment of *Rhodococcus equi* infections in foals [35, 36]. The equine MRSA isolates identified in the present study were sensitive to range of additional antimicrobial classes including chloramphenicol and fluoroquinolones, demonstrating that additional therapeutic options were still available for treating MRSA infections in horses.

The significant association between methicillin resistance and fluoroquinolone resistance in *Staphylococcus* spp. isolates from dogs and cats in this study reflects the observation that a high proportion of Australian MRSP isolates may belong to internationally disseminated fluoroquinolone-resistant clones such as ST71 and ST45 [11, 37], whereas MRSA isolates are likely to belong to ST22-IV [2B], *spa* variable, commonly found in small animal practice veterinarians in Australia [19, 38] and community-acquired infections [39]. A comparative genomics study is currently underway to determine genetic similarity of methicillin-resistant isolates in this study. Comparative genomics represents the most rapid, cost effective and accurate technique for molecular typing including determination of sequence type.

The most important finding from the risk factors study was that particular sites are associated with the risk of a *S*. *pseudintermedius* infection being methicillin-resistant. In agreement with the present study, other studies have also found that isolates from surgical site infections were at higher risk of being resistant to methicillin when compared to other sites [10, 40]. In parallel to the work here, animals that were hospitalised, visited veterinary clinics frequently or had previous antimicrobial treatment were at higher risk for MRSP infections [41]. Compared to studies from 2006, [20, 34] it appears that MRSP infections are becoming increasingly common in veterinary companion animal practice in Australia. The results strongly reinforce the need for veterinarians to place a high priority on implementing infection control procedures, biosecurity and antimicrobial stewardship such as those recommended by the Australian Veterinary Association [42]. Understanding potential factors that lead to emerging resistance may aid in the development of strategies that could curtail the ongoing spread of MRSP within veterinary hospitals.

This study has some limitations. Inclusion of isolates was performed at the convenience and discretion of the animal owners (who would be expected to pay for tests at the primary laboratory), the consulting veterinarian (who may or may not favour sensitivity testing), and the primary laboratory (who may or may not be interested in the study). Consequently, the resulting size and direction of bias in estimates of resistance frequency is difficult to define. Further, the small sample size of methicillin-resistant feline isolates led to wide confidence intervals, limiting our ability to draw statistically significant conclusions on feline isolates. Future studies should therefore focus on achieving a sufficiently large collection of isolates from cats to increase the accuracy of these estimates. Despite these shortfalls, we are unaware of any collection of isolates that is as representative of the Australian population of companion animals, both in terms of size and geographic diversity. Certainly the data presented here surpasses what is currently available elsewhere in the literature [20, 43] and is therefore a useful basis for reviewing prescribing practices for staphylococcal infections in companion animals both in Australia, and more broadly.

## Conclusions

This study shows that antimicrobial resistance is commonly present in the coagulase-positive staphylococci cultured from animal infections in companion animals in Australia. Of greatest concern is the occurrence of moderate levels of MRSP and MRSA, some of which are also resistant to fluoroquinolones. The data provides important baseline measurements for future surveillance and international benchmarking. A strong association of MRSP with surgical site infections in dogs suggests that there could be shortfalls in infection control in animal hospitals. Periodically repeated surveys of this type are crucial for understanding the trends in emergence and dissemination of antimicrobial resistance in companion animals.

## Supporting information

S1 Table Determination of methicillin resistance in *S*. *pseudintermedius* and *S*. *aureus* isolates from dogs, cats and horses in Australia based on phenotypic characteristic and *mecA* PCR. (DOCX)

S2 Table Percentage of resistance to penicillins, cephems and β-lactams/β-lactamase inhibitor combinations before and after confirmation of methicillin resistance status. (DOCX)
