# Supplementary material for: Antimicrobial resistance in coagulase-positive staphylococci isolated from companion animals in Australia: A one year study
Source: PLoS One. 2017 Apr 21;12(4):e0176379. doi: 10.1371/journal.pone.0176379 (PMC5400250; doi:10.1371/journal.pone.0176379)
Supplement: S1 Table — (DOCX) [file pone.0176379.s001.docx]

# **Supporting information**

**S1 Table.** Determination of methicillin resistance in *S. pseudintermedius* and *S. aureus* isolates from dogs, cats and horses in Australia based on phenotypic characteristic and *mecA* PCR.

| Species | Lab ID | Animal origin | IZD (mm) | | MIC (µg/mL) | | Colony appearance in Brilliance^TM^ MRSA2 | *mecA* PCR detection |
| --- | --- | --- | --- | --- | --- | --- | --- | --- |
|  |  |  | FOX | OXA | FOX | OXA |  |  |
| *S. pseudintermedius* | N13/1/238 | Dog | 24 | 0 | 1 | ≥64 | Pale blue | Yes |
| *S. pseudintermedius* | N13/1/359 | Dog | 25 | 0 | 1 | ≥64 | Pale blue- slow growth | Yes |
| *S. pseudintermedius* | N13/1/373 | Dog | 21 | 0 | 4 | ≥64 | Pale blue | Yes |
| *S. pseudintermedius* | N13/1/438 | Dog | 28 | 0 | 1 | ≥64 | Pale blue | Yes |
| *S. pseudintermedius* | N13/1/480 | Dog | 20 | 0 | 16 | ≥64 | Pale blue | Yes |
| *S. pseudintermedius* | N13/1/634 | Dog | 26 | 0 | 2 | ≥64 | Pale blue | Yes |
| *S. pseudintermedius* | N13/1/649 | Dog | 23 | 0 | 2 | ≥64 | Pale blue | Yes |
| *S. pseudintermedius* | N13/1/651 | Dog | 24 | 0 | 2 | ≥64 | Pale blue | Yes |
| *S. pseudintermedius* | N13/1/704 | Dog | 25 | 0 | 2 | ≥64 | Pale blue | Yes |
| *S. pseudintermedius* | N13/1/748 | Dog | 22 | 0 | 4 | ≥64 | Pale blue | Yes |
| *S. pseudintermedius* | N13/1/839 | Dog | 23 | 0 | 2 | ≥64 | Pale blue | Yes |
| *S. pseudintermedius* | N13/4/115 | Dog | 24 | 0 | 8 | ≥64 | Pale blue | Yes |
| *S. pseudintermedius* | N13/4/121 | Dog | 25 | 10 | 2 | ≥64 | Pale blue | Yes |
| *S. pseudintermedius* | N13/4/123 | Dog | 24 | 0 | 4 | ≥64 | Pale blue | Yes |
| *S. pseudintermedius* | N13/4/25 | Dog | 33 | 0 | 2 | ≥64 | Pale blue- slow growth | Yes |
| *S. pseudintermedius* | N13/4/39 | Dog | 31 | 15 | 1 | ≥64 | Pale blue- slow growth | Yes |
| *S. pseudintermedius* | N13/4/52 | Dog | 32 | 0 | 2 | ≥64 | Pale blue | Yes |
| *S. pseudintermedius* | N13/4/75 | Dog | 30 | 10 | 1 | ≥64 | Pale blue- slow growth | Yes |
| *S. pseudintermedius* | N13/4/94 | Dog | 23 | 0 | 8 | ≥64 | Pale blue | Yes |
| *S. pseudintermedius* | Q13/1/190 | Dog | 20 | 0 | 8 | ≥64 | Pale blue | Yes |
| *S. pseudintermedius* | Q13/1/243 | Dog | 26 | 0 | 2 | ≥64 | Pale blue | Yes |
| *S. pseudintermedius* | Q13/1/294 | Cat | 14 | 0 | 32 | ≥64 | Pale blue | Yes |
| *S. pseudintermedius* | Q13/1/311 | Dog | 29 | 0 | 0.5 | ≥64 | Pale blue | Yes |
| *S. pseudintermedius* | Q13/1/317 | Dog | 23 | 0 | 8 | ≥64 | Pale blue | Yes |
| *S. pseudintermedius* | Q13/1/326 | Dog | 28 | 0 | 8 | ≥64 | Pale blue | Yes |
| *S. pseudintermedius* | Q13/3/18 | Dog | 26 | 0 | 4 | ≥64 | Pale blue | Yes |
| *S. pseudintermedius* | Q13/3/24 | Dog | 26 | 0 | 2 | ≥64 | Pale blue | Yes |
| *S. pseudintermedius* | V13/2/18 | Dog | 22 | 0 | 4 | ≥64 | Pale blue | Yes |
| *S. pseudintermedius* | V13/2/125 | Dog | 33 | 16 | 4 | ≥64 | Pale blue | Yes |
| *S. pseudintermedius* | V13/2/133 | Dog | 29 | 0 | 2 | ≥64 | Pale blue | Yes |
| *S. pseudintermedius* | V13/2/140 | Dog | 32 | 0 | 2 | ≥64 | Pale blue | Yes |
| *S. pseudintermedius* | V13/2/152 | Dog | 26 | 0 | 8 | ≥64 | Pale blue | Yes |
| *S. pseudintermedius* | V13/2/191 | Dog | 28 | 0 | 1 | ≥64 | Pale blue | Yes |
| *S. pseudintermedius* | V13/2/393 | Dog | 25 | 0 | 8 | ≥64 | Pale blue | Yes |
| *S. pseudintermedius* | V13/2/407 | Dog | 28 | 0 | 1 | ≥64 | Pale blue | Yes |
| *S. pseudintermedius* | V13/2/413 | Dog | 21 | 0 | 1 | ≥64 | Pale blue | Yes |
| *S. pseudintermedius* | V13/2/440 | Dog | 29 | 0 | 0.5 | ≥64 | Pale blue | Yes |
| *S. pseudintermedius* | V13/2/441 | Dog | 30 | 0 | 1 | ≥64 | Pale blue | Yes |
| *S. pseudintermedius* | V13/2/475 | Dog | 30 | 0 | 1 | ≥64 | Pale blue | Yes |
| *S. pseudintermedius* | V13/2/488 | Dog | 30 | 0 | 1 | ≥64 | Pale blue | Yes |
| *S. pseudintermedius* | V13/2/52 | Dog | 18 | 0 | 32 | ≥64 | Pale blue | Yes |
| *S. pseudintermedius* | V13/2/92 | Cat | 30 | 12 | 2 | ≥64 | Pale blue | Yes |
| *S. pseudintermedius* | W13/1/11 | Dog | 24 | 0 | 4 | ≥64 | Pale blue | Yes |
| *S. pseudintermedius* | N13/1/103 | Dog | 23 | 0 | 8 | 32 | Pale blue | Yes |
| *S. pseudintermedius* | N13/1/304 | Dog | 12 | 0 | 32 | 32 | White blue | Yes |
| *S. pseudintermedius* | N13/1/580 | Dog | 28 | 10 | 4 | 32 | Pale blue | Yes |
| *S. pseudintermedius* | N13/1/77 | Dog | 27 | 17 | 2 | 32 | Pale blue | Yes |
| *S. pseudintermedius* | Q13/1/35 | Dog | 25 | 0 | 2 | 32 | Pale blue | Yes |
| *S. pseudintermedius* | V13/2/193 | Dog | 25 | 0 | 2 | 32 | Pale blue | Yes |
| *S. pseudintermedius* | V13/6/5 | Dog | 30 | 0 | 0.25 | 32 | Pale blue | Yes |
| *S. pseudintermedius* | W13/1/12 | Dog | 23 | 12 | 4 | 32 | Pale blue | Yes |
| *S. pseudintermedius* | V13/2/173 | Dog | 30 | 0 | 2 | 16 | Pale blue | Yes |
| *S. pseudintermedius* | V13/2/83 | Dog | 29 | 0 | 2 | 16 | Pale blue | Yes |
| *S. pseudintermedius* | V13/2/220 | Dog | 33 | 0 | 1 | 8 | Pale blue- slow growth | Yes |
| *S. pseudintermedius* | N13/4/21 | Dog | 29 | 16 | 0.5 | 4 | Pale blue | Yes |
| *S. pseudintermedius* | Q13/1/21 | Dog | 26 | 0 | 1 | 4 | Pale blue | Yes |
| *S. pseudintermedius* | Q13/1/219 | Dog | 30 | 13 | 1 | 4 | Pale blue | Yes |
| *S. pseudintermedius* | V13/2/227 | Dog | 26 | 10 | 1 | 4 | Pale blue- slow growth | Yes |
| *S. pseudintermedius* | V13/2/242 | Dog | 30 | 0 | 1 | 4 | Pale blue | Yes |
| *S. pseudintermedius* | V13/2/194 | Dog | 30 | 0 | 1 | 2 | Pale blue | Yes |
| *S. pseudintermedius* | V13/2/229 | Dog | 31 | 20 | 32 | 2 | Pale blue | Yes |
| *S. pseudintermedius* | V13/6/4 | Dog | 25 | 0 | 8 | 2 | Pale blue | Yes |
| *S. pseudintermedius* | V13/6/7 | Dog | 27 | 0 | 1 | 2 | Pale blue | Yes |
| *S. pseudintermedius* | N13/1/307 | Dog | 27 | 14 | 0.5 | 1 | Pale blue- slow growth | Yes |
| *S. pseudintermedius* | N13/1/386 | Dog | 26 | 0 | 0.5 | 1 | Pale blue- slow growth | Yes |
| *S. pseudintermedius* | N13/1/421 | Dog | 28 | 10 | 0.5 | 1 | Pale blue | Yes |
| *S. pseudintermedius* | N13/1/446 | Dog | 25 | 0 | 0.5 | 1 | Pale blue | Yes |
| *S. pseudintermedius* | N13/1/616 | Dog | 27 | 20 | 0.5 | 0.5 | Pale blue | Yes |
| *S. pseudintermedius* | N13/1/700 | Dog | 28 | 15 | 0.5 | 0.5 | Pale blue- slow growth | Yes |
| *S. pseudintermedius* | N13/4/19 | Dog | 32 | 20 | 0.5 | 0.5 | Pale blue- slow growth | Yes |
| *S. pseudintermedius* | V13/2/16 | Dog | 32 | 21 | 1 | 0.5 | Pale blue- slow growth | Yes |
| *S. pseudintermedius* | V13/2/379 | Dog | 0 | 0 | 0.25 | 0.5 | Pale blue- slow growth | Yes |
| *S. pseudintermedius* | W13/1/4 | Dog | 27 | 20 | 1 | 0.5 | Pale blue | Yes |
| *S. pseudintermedius* | W13/1/5 | Dog | 12 | 14 | 0.5 | 0.5 | Pale blue- slow growth | Yes |
| *S. pseudintermedius* | N13/1/317 | Dog | 25 | 0 | 2 | ≥64 | Pale blue | No |
| *S. pseudintermedius* | V13/2/42 | Dog | 0 | 0 | ≥64 | 8 | No growth | No |
| *S. pseudintermedius* | S13/1/74 | Dog | 34 | 27 | 4 | 2 | No growth | No |
| *S. pseudintermedius* | N13/4/59 | Dog | 32 | 19 | 0.5 | 1 | Pale blue- slow growth | No |
| *S. pseudintermedius* | N13/1/466 | Cat | 25 | 17 | 1 | 0.5 | No growth | No |
| *S. pseudintermedius* | N13/1/627 | Dog | 33 | 19 | 0.25 | 0.5 | Pale blue | No |
| *S. pseudintermedius* | N13/1/677 | Dog | 30 | 19 | 2 | 0.5 | No growth | No |
| *S. aureus* | N13/1/17 | Cat | 8 | 0 | ≥64 | ≥64 | Blue | Yes |
| *S. aureus* | N13/1/382 | Cat | 10 | 0 | ≥64 | ≥64 | Blue | Yes |
| *S. aureus* | N13/1/648 | Horse | 12 | 0 | ≥64 | ≥64 | Blue | Yes |
| *S. aureus* | N13/1/715 | Horse | 7 | 0 | ≥64 | ≥64 | Blue | Yes |
| *S. aureus* | Q13/1/141 | Dog | 0 | 0 | ≥64 | ≥64 | Blue | Yes |
| *S. aureus* | Q13/1/145 | Dog | 0 | 0 | ≥64 | ≥64 | Blue | Yes |
| *S. aureus* | Q13/1/305 | Dog | 12 | 0 | ≥64 | 32 | Blue | Yes |
| *S. aureus* | V13/2/426 | Dog | 11 | 0 | ≥64 | ≥64 | Blue | Yes |
| *S. aureus* | V13/2/458 | Horse | 12 | 0 | ≥64 | ≥64 | Blue | Yes |
| *S. aureus* | N13/1/396 | Horse | 12 | 0 | 32 | ≥64 | Blue | Yes |
| *S. aureus* | N13/1/408 | Horse | 13 | 0 | 32 | ≥64 | Blue | Yes |
| *S. aureus* | N13/4/96 | Horse | 16 | 0 | 32 | 32 | Blue | Yes |
| *S. aureus* | V13/2/439 | Dog | 16 | 18 | 32 | ≥64 | Blue | Yes |
| *S. aureus* | Q13/1/325 | Dog | 16 | 0 | 16 | 32 | Blue | Yes |
| *S. aureus* | V13/6/23 | Cat | 17 | 10 | 32 | 4 | Blue- small colonies | No |

*IZD- inhibition zone diameter; MIC- minimum inhibitory concentration; FOX- cefoxitin; OXA- oxacillin. Colony appearance on Brilliance™ Agar was described as slow growth if colonies of typical blue or pale blue appearance were observed after 48 hrs instead of the typical 24hrs incubation. Isolates shaded in grey were negative for the *mecA* PCR and for the purposes of this paper- classified as methicillin-susceptible *S. pseudintermedius*. A single *S. aureus* isolate from a cat (V12/6/23) that was negative for *mecA* PCR- was subsequently shown to contain a *mecC* element [21].
