# Supplementary material for: Antimicrobial resistance in coagulase-positive staphylococci isolated from companion animals in Australia: A one year study
Source: PLoS One. 2017 Apr 21;12(4):e0176379. doi: 10.1371/journal.pone.0176379 (PMC5400250; doi:10.1371/journal.pone.0176379)
Supplement: S2 Table — (DOCX) [file pone.0176379.s002.docx]

# **Supporting Information**

**S2 Table.** Percentage of resistance to penicillins, cephems and β-lactams/β-lactamase inhibitor combinations before and after confirmation methicillin resistance status.

| Antimicrobial agent | No. of resistant isolates determined by MIC breakpoints | No. of isolates reported as resistant determined by MIC breakpoints and methicillin resistance status |
| --- | --- | --- |
| *S. pseudintermedius* (dogs n=616) |  |  |
| Amoxicillin-clavulanate | 231 (37.5 %) | 239 (38.8 %) |
| Cefovecin | 81 (13.1 %) | 86 (14 %) |
| Cefoxitin | 71 (11.5 %) | 84 (13.6%) |
| Ceftriaxone | 79 (12.8 %) | 85 (13.8 %) |
| Cephalothin | 83 (13.5 %) | 93 (15.1 %) |
| Oxacillin | 78 (12.7 %) | 78 (12.7%) |
|  |  |  |
| *S. pseudintermedius* (cats n=13) |  |  |
| Amoxicillin-clavulanate | 7 (53.8 %) | 7 (53.8 %) |
| Cefovecin | 2 (15.4%) | 2 (15.4%) |
| Cefoxitin | 3 (23.1 %) | 3 (23.1 %) |
| Ceftriaxone | 3 (23.1 %) | 3 (23.1 %) |
| Cephalothin | 3 (23.1 %) | 3 (23.1 %) |
| Oxacillin | 3 (23.1 %) | 3 (23.1 %) |
|  |  |  |
| *S. aureus* (horses n=53) |  |  |
| Amoxicillin-clavulanate | 25 (47.2 %) | 25 (47.2 %) |
| Cefovecin | 7 (13.2 %) | 7 (13.2 %) |
| Cefoxitin | 6 (11.3 %) | 6 (11.3 %) |
| Ceftriaxone | 7 (13.2 %) | 7 (13.2 %) |
| Cephalothin | 6 (11.3 %) | 7 (13.2 %) |
| Oxacillin | 6 (11.3 %) | 6 (11.3 %) |
|  |  |  |
| *S. aureus* (dogs n=47) |  |  |
| Amoxicillin-clavulanate | 27 (54.7 %) | 27 (54.7 %) |
| Cefovecin | 7 (14.9 %) | 7 (14.9 %) |
| Cefoxitin | 6 (12.8 %) | 6 (12.8 %) |
| Ceftriaxone | 6 (12.8 %) | 6 (12.8 %) |
| Cephalothin | 6 (12.8 %) | 6 (12.8 %) |
| Oxacillin | 6 (12.8 %) | 6 (12.8 %) |
|  |  |  |
| *S. aureus* (cats n=17) |  |  |
| Amoxicillin-clavulanate | 10 (58.8 %) | 10 (58.8 %) |
| Cefovecin | 3 (17.6%) | 3 (17.6%) |
| Cefoxitin | 3 (17.6%) | 3 (17.6%) |
| Ceftriaxone | 3 (17.6%) | 3 (17.6%) |
| Cephalothin | 2 (11.8%) | 3 (17.6%) |
| Oxacillin | 3 (17.6%) | 3 (17.6%) |

*Shaded areas show changes of percentage after methicillin resistance status.
